# The Communicability of Graphical Alternatives to Tabular Displays of Statistical Simulation Studies

**DOI:** 10.1371/journal.pone.0027974

**Published:** 2011-11-23

**Authors:** Alex R. Cook, Shanice W. L. Teo

**Affiliations:** 1 Saw Swee Hock School of Public Health, National University of Singapore, Singapore, Republic of Singapore; 2 Department of Statistics and Applied Probability, National University of Singapore, Singapore, Republic of Singapore; 3 Program in Health Services and Systems Research, Duke-NUS Graduate Medical School Singapore, National University of Singapore, Singapore, Republic of Singapore; University of Modena and Reggio Emilia, Italy

## Abstract

Simulation studies are often used to assess the frequency properties and optimality of statistical methods. They are typically reported in tables, which may contain hundreds of figures to be contrasted over multiple dimensions. To assess the degree to which these tables are fit for purpose, we performed a randomised cross-over experiment in which statisticians were asked to extract information from (i) such a table sourced from the literature and (ii) a graphical adaptation designed by the authors, and were timed and assessed for accuracy. We developed hierarchical models accounting for differences between individuals of different experience levels (under- and post-graduate), within experience levels, and between different table-graph pairs. In our experiment, information could be extracted quicker and, for less experienced participants, more accurately from graphical presentations than tabular displays. We also performed a literature review to assess the prevalence of hard-to-interpret design features in tables of simulation studies in three popular statistics journals, finding that many are presented innumerately. We recommend simulation studies be presented in graphical form.

## Introduction

Simulation studies are vital in assessing statistical methods. They allow frequency properties or other criteria to be compared for competing methods, thus providing evidence to facilitate the establishment of new methods and, subsequently, their potential application to problems in science, medicine and the social sciences. In evaluating methodologies, simulation studies: (i) provide a cost-effective way to quantify potential performance for a large range of scenarios, spanning different combinations of sample sizes and underlying parameters, (ii) allow average performance to be estimated under repeat Monte Carlo sampling and (iii) facilitate comparison of estimates against the “true” system underlying the simulations, none of which is really achievable via genuine applications, as gratifying as those are.

As a consequence, many statistics journals brim with simulation studies. A challenge to the authors of such papers is deciding how to present simulated results. Typically, at least two methods are contrasted with respect to several objective functions, over a design space of at least one dimension (and often several); it is this complexity that confounds simple presentation. The usual approach is to present the simulation study in one or more tables.

It has long been pointed out that tabular displays of data (and by extension simulated data) can be difficult to interpret if constructed badly, with the task of taking information from a table having famously been likened to that of *extracting sunlight from a cucumber* [1] (as cited in [2]). Ehrenberg [3] and discussants of his and Mahon's [4] papers note “commonsense” advice going back to the 1910s on how to present tabular information. Ehrenberg [3] and, later, Wainer [2] outline a series of guidelines to present quantitative information numerately: round heavily (they recommend to two effective digits), provide anchoring via averages, make primary comparisons vertically, use effective ordering and good use of space. Ehrenberg [3] argues convincingly that when a reader finds a table of figures hard to understand, even when told what to look for (his “weak criterion” for a good table), the fault lies with the producer of the table. As in good writing, the onus is on the author to facilitate the reader's comprehension. Our experience has been that many of the guidelines presented by Ehrenberg [3] and Wainer [2] are frequently broken by statisticians when presenting their own work in papers, seminars or conferences, and that many journals adopt tabular formatting requirements that do not abide by these guidelines.

In presenting simulation studies, authors have a choice of media: text, table or graph. Graphs have been recommended to display relationships or comparisons, tables for values [4], [5], but a review of an issue of the *Journal of the American Statistical Association* suggested that most tables presented therein were used for comparisons [5], a task to which they are suboptimal. In their aforementioned review, Gelman et al. [5] demonstrated that it is possible to create graphical variants of tables that are as compact as the table they might replace for a range of examples. A recent review found that, even in the *Journal of Computational and* Graphical *Statistics*, tables accounted for a third of all displays over the period 2005 to 2010 [6].

Several explanations for the preference for tables over graphs have recently been suggested [7]–[12]. We believe that one further reason why the adoption of graphical displays of simulation studies is non-universal is the relative shortage of quantitative evidence in favour of graphs (or, contrariwise, tables), especially for constructions as complex as in multidimensional simulation studies. Previous research has been carried out primarily on small displays (e.g. refs. [13]–[16]) and as a consequence it is unclear how relevant previous findings (which have been mixed, with some supporting graphs and others tables, see the summary in ref. [5]) are to the problem of displaying large simulation study results. In addition, most have focused on the display of actual data, whereas the numerical results of simulation studies typically consist of more abstract performance metrics to which, being less grounded in our everyday experiences, it may be harder to relate. At the same time, most previous studies have generally abided by the rules of good tabular design (though not always of good graphical design, as espoused by Tufte [17] and Cleveland [18], [19] *inter alia*) and therefore are aspirational rather than descriptive of the current state of numerical information in methodological statistics.

To assess the relative communicability of tabular and graphical displays of large simulation studies, we designed and performed a randomised cross-over experiment to compare quantitatively the speed and accuracy at which information could be extracted from graphical or tabular displays by statisticians with varying levels of experience in statistical research. We analysed the results using an hierarchical model accounting for the heterogeneity in intrinsic difficulty between a set of representative tables selected from the literature and matching graphs designed by the authors, and between individual differences in speed and accuracy. This we fitted in the Bayesian paradigm. We also performed a systematic review of three statistical journals–*the Journal of the American Statistical Association*, *the Annals of Statistics* and *Statistica Sinica*–to assess the prevalence of bad design features in tabular displays of simulation studies.

## Materials and Methods

### Ethics statement

Participants in the experiment provided written informed consent and were able to withdraw from the study at any point before the anonymisation of their data. The study design was approved by the Institutional Review Board of the National University of Singapore.

### Review of literature

We investigated the prevalence of bad design features in tabular displays of simulation studies via a systematic survey of three statistical journals: two of the most prestigious, the *Annals of Statistics* and the *Journal of the American Statistical Association*, and another, *Statistica Sinica*, which is well regarded. We limited attention to tables portraying simulation studies, and performed a census of all such tables from all issues of these three journals published in the year 2009. We chose five criteria with reference to the discussion by Ehrenberg [3] and Wainer [2] as a means of assessing how well constructed these tables were, these being

- the number of entries (the fewer, the easier to comprehend),
- the maximum number of (non-leading zero) digits presented (ditto),
- binary indicators coding the alignment of decimal places (aligned is preferred),
- vertical versus horizontal comparisons (vertical is preferred), and
- the presence of parenthesis (often [unnecessary] visual clutter).

### Experimental protocol

Twenty volunteers participated in the randomised cross-over experiment. Ten were undergraduate students near the end of a BSc in statistics at the National University of Singapore (NUS), with some (limited) exposure to statistical methodological research. Another ten were either current postgraduate research students pursuing a PhD in statistics or were faculty members with research interests in statistics or applied probability and holding a PhD in statistics or an allied discipline, again at NUS. The dichotomisation into two groups was to allow us to assess the notion that statistical research experience might influence the perceived relative difficulty between tabular and graphical displays.

We selected six tables of simulation studies from the literature (refs. [20]–[25]) to be one half of the experimental stimuli. Our intention was that these would cover the most common types of simulation study displays, and to this end they were selected purposefully, not randomly. The tables selected and a quantitative summary of their complexity are presented in Table 1.

**Table 1:** Details of the six tables taken from the literature used as stimuli in the cross-over experiment.

Source table	Source ref.	Precision	Number of methods	Comparison dimensionality
5.1	[20]	4	6	3
4	[21]	3	3	3
3	[22]	3	3	3
1	[23]	3	5	2
3	[24]	3	5	1
1	[25]	3	11	4

The other stimuli–graphical adaptations of the tables below, designed by the authors– appear in Supporting Information S1. The maximum precision in non-leading zero digits, the number of methods being compared, and the number of other dimensions (e.g. sample size, or effect size) along which comparisons could be made, are tabulated.

For each of these six tables we created a matching graph, using the grid package [26] within the R statistical environment [27]. The package grid allows the user the power to design graphs to the minutest detail, and probably attains the current apex of statistical graphics. We used grid to maximise the information content of the six matching graphs by controlling spacing, axis labelling and colouring of graph elements. The resulting graphs are presented in Supporting Information S1.

For all six table/graph pairs, we then wrote a set of five questions which participants were to answer as quickly and accurately as possible. The questions (provided in Supporting Information S1) were written to emulate the sort of questions the authors ask when looking at tables of simulation studies, for instance: *which method has the least accurate estimated coverage probability in all cases considered?* and *which test usually has the lowest power?* Many of the questions were rather difficult to answer since they required comparing numbers across several dimensions at one time–a common challenge in trying to understand simulation studies. All questions were constructed to allow an answer that could be marked correct or not, although this constraint may have lessened the realism of the experiment.

The experimental protocol was as follows. Each participant was allocated in advance one graph and one table, drawn uniformly randomly from all 30 pairs of non-matching graphs and tables using a pseudo-random number generator. Participants sat in a quiet room with one or both of the authors. After providing written informed consent, they were presented with one stimulus, either a graph or a table (the ordering selected pseudo-randomly *a priori*), and some background information on the nature of the research problem tackled in the paper containing the original table. They were given as long as they wished to read this. They were then presented with the associated list of five questions for that stimulus and the time taken to answer each question recorded. After completing the five questions, the process was repeated using the other stimulus. Afterwards, the authors assigned a binary correctness score to each answer using a pre-designed rubric. The ten times in seconds and scores were then entered anonymously into a database along with the experience level of the participant (under- or post-graduate) and a categorical indicator of the stimuli used. The data are provided in Supporting Information S2 and S3.

No participants elected to withdraw from the study.

### Data analysis

To account for the structured nature of the experiment–repeat observations from different combinations of graphs/tables and study participants of two different experience levels–we use multilevel models (see [28] for example) fit in the Bayesian paradigm for both timing and accuracy data. The Bayesian approach is attractive here for several reasons: (i) the model has to have multiple levels to represent the structure of the data, and Bayesian methods are particularly suited to such models; (ii) some elements of the data hierarchy have small samples–ten individuals per experience level, six stimuli–and hence any assumption of having reached asymptotic normality has to be suspect; and (iii) a Bayesian approach readily allows estimates and uncertainty intervals of functions of the parameters to be obtained. For the timing data, we work with the natural logarithm of the time in seconds, and take the mean log-time for the 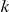
th measurement for the 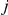
th treatment on the 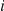
th individual (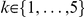, 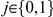, 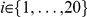) to be: 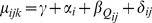
(1)


where

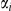
 captures the speed of individual 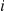 and is assumed to come from a normal distribution with standard deviation 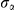 and, for undergraduate participants, mean zero, while for postgraduate participants, the mean was the free parameter, 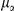;

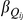
 quantifies the difficulty of the table or graph 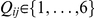 selected randomly from the bank for which 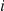 is answering questions, and is assumed to come from a mean zero normal distribution with standard deviation 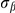;

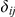
 captures the effect on speed of being presented a table (for 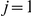) rather than a graph (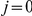); allowing an interaction with the experience of individual 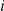 we have 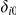 fixed to 0, 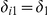 for undergraduates, and 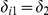 for postgraduates, the latter two free parameters.

Note that some parameters described above are set to zero for the sake of identifiability. We assume homoskedasticity of the distribution of log-times. All parameters are assigned non-informative prior distributions (see Supporting Information S1 for details).

For the accuracy measurements, a very similar model was fitted, using a Bernoulli distribution for the classification of each answer as correct or not, with the logit of the probability of an accurate answer replacing the mean log-time in equation (1) and the assumption of homoskedasticity being dropped.

The models were fitted using Markov chain Monte Carlo sampling [29]–[32] in OpenBUGS version 3.1.1 [33] with four samplers with over-dispersed starting points and 100 000 iterations following a burn-in of 20 000 and every 10th iteration retained for later analysis. Convergence was assessed using the Gelman–Rubin [34] and Brooks–Gelman [35] diagnostics in the CODA package [36] for R [27], as well as by visual assessment of the resulting trace plots.

The retained output was exported to R via CODA for post-processing, involving converting back from the logarithmic or logistic scale to the original data scale to facilitate interpretation. Estimates are accompanied by 95% (credible) intervals (95%I).

## Results

### Review of literature

**Figure 1:**
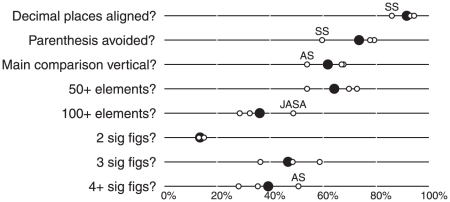
Prevalence of undesirable design features in tabular displays in statistics journals. All tables containing results of simulation studies in articles published in the *Journal of the American Statistical Association* (JASA), *Annals of Statistics* (AS) and *Statistica Sinica* (SS) during the year 2009 comprise the population reviewed. The proportion satisfying various criteria are marked: for the first three, the higher the proportion the better, while smaller tables with fewer significant figures (sig figs) are preferred. Overall proportions are indicated with a solid circle, within journal proportions by a hollow circle. For each criterion, if one journal did notably worse than the others, its proportion is labelled.

From 313 articles published in the three focus journals in 2009, we identified 184 (59%) that contained some form of simulation study. A representation of the prevalence of undesirable design features is presented in Figure 1. The focus journals for the most part succeed in avoiding visual problems that can be addressed at the type-setting stage, with most tables having decimal places aligned (91%), thereby enabling the reader to determine the order of magnitude of numbers at a glance, and avoiding the use of parenthesis (74%), which add unnecessary visual clutter.

Several more inherent obstacles to interpretation remain. Some 38% of tables are set up so that the main direction along which comparisons are made is horizontal, rather than vertical. Wainer [2] and Ehrenberg [3] demonstrate why this is more challenging for the reader to understand. Many tables are burdened with a great volume of numbers, with 36% having more than 100 numbers and some many more (the average is almost exactly 100). The final metric we assessed was the number of digits presented, and here, again, the advice from the literature is for the most part unheeded: 86% of tables tabulate three or more digits, and 39% four or more. There seems little reason for such spurious accuracy, especially since many of these numbers were generated from small Monte Carlo samples, so even if this degree of purported accuracy were helpful to the reader (it isn't), in most situations it would not be warranted on statistical grounds.

There were some differences between the three journals that may interest the dedicated reader: *Statistica Sinica* generally did worse on typographic issues, tables in the *Journal of the American Statistical Association* were the most voluminous, while tables in the *Annals of Statistics* were more likely to be poorly oriented and to have unhelpful overprecision.

### Cross-over experiment

**Figure 2:**
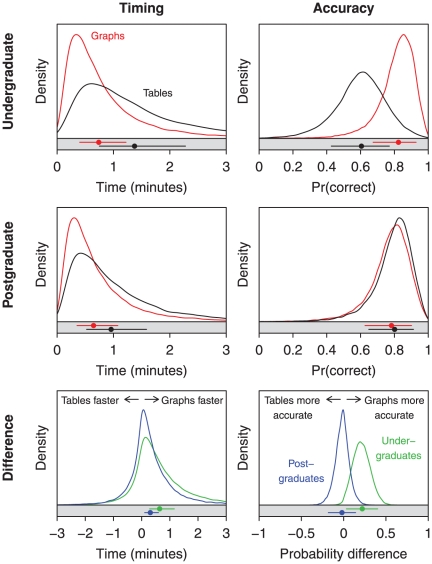
Comparison of speed and accuracy at which information can be drawn from tables and figures in randomised cross-over experiment, by experience level. Distributions in white panels account for parametric uncertainty and variability between individuals and table-graph pairs, and are estimated using standard kernel density estimation from MCMC samples. Distributions in grey panels are for an average individual, an average graph and account only for parameter uncertainty; posterior mean and 95% intervals are plotted. Left: timing in minutes per question. Right: probability of reporting a correct answer. Top: undergraduate statistics students, by medium. Middle: PhD candidates or faculty members, by medium. Bottom: difference between graph and table speed and accuracy of information extraction, by experience.

The results of the experiment showed clear evidence that information could be extracted quicker from graphs than tables (Figure 2, left), with the overall average difference in times of 27s (95%I: 14 s, 47 s). Interestingly, the difference between the two media was more pronounced for undergraduate participants–i.e. those with generally less experience of mathematical statistical research–with undergraduates needing around an additional 38 s to answer a question using a table than the equivalent graph (95%I: 17 s, 70 s), i.e. almost double the time, while postgraduates required an additional 19 s (95%I: 7 s, 36 s), around only a 50% increase. Variability within experience groups was low, though present, but variability between the time needed, presumably a reflexion of differences in their inherent difficulty, to extract information from some of the table–graph pairs was substantial.

There was a similar interaction between statistical research experience and the accuracy with which information could be extracted from graphical and tabular displays of simulation studies (Figure 2, right). There was no discernible difference between the accuracy of answers derived from tables (80%, 95%I [65,91]%) or graphs (78%, [63,90]%) for the more experienced participants, and the accuracy by which undergraduates could extract information from a graph (82%, [67,93]%) was about the same as that of postgraduates faced with the same graph, but undergraduate participants were markedly less accurate at taking information from tables (60%, [43,77]%; difference 21%, [2,42]%).

The “catch up” effect that allowed less experienced participants to extract information from a graph at a similar speed and accuracy as more experienced participants was also manifested in comparisons between graphical displays read by undergraduates and tabular ones read by postgraduates. There was no discernable difference in accuracy between these experience-stimuli pairs (difference 2%, [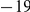,14]%) while there was marginal evidence that the undergraduate armed with a graph could parse it quicker than a table perusing postgraduate (difference 13s, [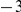,33]s).

## Discussion

Our results strongly suggest that, to the participants in this experiment, graphical displays provided a more efficient way to communicate the findings of simulation studies than tables. Our readers could parse the graphical equivalent demonstrably faster, and for our undergraduate participants, the graphical medium provided a more accurate understanding of the results of the simulation study (the relatively small sample size prevented us from corroborating if postgraduate participants also had improved accuracy). Had the authors of the source tables switched to a graphical display, less experienced readers would have been able to read and understand the display as quickly and accurately as a reader with postgraduate experience of statistical research, while at the same time allowing the experienced reader to understand the results of the simulation study with less effort.

In reviewing all papers published in three high profile mathematical statistics journals in 2009, we found that many authors continue to present simulation studies in tabular form, and that the prevalence remains high of several characteristics that may impair the ability to understand tables effectively. Such tables are often burdened with too many numbers to be easily understood, are often displayed to too much precision to make sense of or even to make sense (when one considers the often small Monte Carlo sample size from which each element in the table has been derived and the concomitant standard errors), and are often oriented opposite the simplest–vertical–direction in which to compare related numbers. The two design features we quantified that are most easily addressed at the typesetting stage–alignment of decimal places and the preference for unbracketed numbers where possible–were also the two features for which the three journals most consistently adhered to the advice of Ehrenberg [3] and Wainer [2], suggesting that authors, not journals, are most culpable for poorly designed tables of simulation studies.

The arguments in favour of graphical presentation of simulation studies are multifold. Graphs provide an easier way to obtain an holistic view of the study, make more forceful statements, and allow specific comparisons more readily, the latter particularly important when multiple methods are contrasted across varying sample sizes and underlying conditions. Wainer [2] suggests four purposes for numerical information, namely *exploration*, *communication*, *storage* and *decoration*. Our expectation is that of these, only *communication* and *decoration* are relevant for simulation studies in research reports: the *exploration* phase has presumably already been completed by the authors (though graphs may help here, too [37]), who are now seeking to communicate their findings, while *storage* of cheap Monte Carlo “data” seems irrelevant. Since even badly designed graphs are more eye-catching than ranks of numerical simulation data, we believe that authors who use the table for *decoration* would be best to switch to a graph. This leaves *communication*, but the results of our experiment support the argument that this, too, may best be achieved by some kind of graphic.

We anticipate that switching to graphical displays would permit useful structural changes to simulation studies: increasing the number of design points, such as simulated sample sizes, to give a broader picture of the difference between methodologies, and creating more natural opportunities to present uncertainty in Monte Carlo estimates (e.g. via confidence intervals), which currently is, oddly, often neglected in tabular displays. A related curious omission from many tables of simulation studies is a formal statistical analysis of the (simulation) experiment's results; we believe that the results of such analyses of simulated output could gainfully be presented alongside or in place of a graphical rendition of the raw simulation output.

There are of course several limitations to the experimental design that may inhibit the generality of our findings. Study participants were selected from a single environment, namely students and staff of the National University of Singapore, and may consequently differ in subtle ways from consumers of statistical simulation studies in other settings. We purposefully selected individuals with a statistical background, believing them to be the target audience of papers containing statistical simulation studies, and it is unclear to what extent readers with different backgrounds–in particular, with little theoretical statistical training–would differ from the study participants. A further possible limitation is the risk of idiosyncrasies in the particular tables selected and our graphical adaptations thereof: we invested quite a bit of effort in devising suitable graphical presentations of the tables we found, and it is possible (though we hope unlikely) that the authors of the original tables created them without a similar investment. As a reviewer points out, one cannot fairly compare the compositions of Mozart and Lennon when one is performed by a school band and the other a philharmonic orchestra. A fairer assessment of the relative merits of tables and graphs might result from a competition between proponents of the two sides, similar to the recent graphic competition held by *Chance* magazine [38]. A further limitation is that the performance metrics of time and accuracy related to an artificial task, namely of providing answers to questions that could be quantified as correct or not, though in their natural environment, numerical displays may induce questions that do not yield simplistic, correct/incorrect answers.

The experiment we performed considered design characteristics of tables and graphs at an aggregated level, and thus do not shed light on particular elements of graphical or tabular displays of simulation studies. Although there already exist useful guidelines on how best to design tabular displays [2], [3], [6], there is scope for future work to elicit quantitatively the effect of some of the design features in Figure 1 –such as the volume of numbers in tables or the precision to which they are presented–on the ability of readers to parse information. Such research would allow statistical simulation studies to be designed more efficiently, from the perspective of presenting the results in an intelligible fashion to other researchers, and would also indicate how general the results of this study are. Although we have focused on displays of statistical simulation studies, we expect that the efficiency of displays of data in other fields–such as medicine, in which tables are even more widespread [6] –would be amenable to future research.

In summary, this study demonstrated that many tables of simulation studies containing undesirable features may be found in the statistical literature, and that–among our study participants at least–these features make the studies harder to understand than a well-constructed graphical equivalent, and may lead to erroneous conclusions for non-experts. Over 30 years ago, Ehrenberg opened his landmark treatise on the rudiments on numeracy thus: *Many tables of data are badly presented* [3]. It is our opinion that this is still the case and that most, if not all, results of statistical simulation studies should be presented in graphical form.

## Supporting Information

Supporting Information S1 **Appendices.** Appendix 1 contains details of the cross-over experiment, namely the background information provided to participants, the graphical stimuli, and questions asked. Appendix 2 contains details of the prior distributions used in the analysis.
(PDF)

Supporting Information S2 **Data from cross-over experiment.**
(CSV)

Supporting Information S3 **Readme file for Supporting Information S2.**
(TXT)
