# Supplementary material for: The Communicability of Graphical Alternatives to Tabular Displays of Statistical Simulation Studies
Source: PLoS One. 2011 Nov 23;6(11):e27974. doi: 10.1371/journal.pone.0027974 (PMC3223202; doi:10.1371/journal.pone.0027974)
Supplement: Supporting Information S1 — Appendices. Appendix 1 contains details of the cross-over experiment, namely the background information provided to participants, the graphical stimuli, and questions asked. Appendix 2 contains details of the prior distributions used in the analysis. (PDF) [file pone.0027974.s001.pdf]

The communicability of graphical alternatives to tabular displays of statistical simulation studies

Alex R Cook[1,2,3*], Shanice W L Teo[2]
1 Saw Swee Hock School of Public Health, National University of Singapore, Singapore, Republic of Singapore
2 Department of Statistics and Applied Probability, National University of Singapore, Singapore, Republic of Singapore
3 Program in Health Services and Systems Research, Duke-NUS Graduate Medical School Singapore, National University of Singapore, Singapore, Republic of Singapore
∗ **E-mail: alex.richard.cook@gmail.com**

Appendix 1: details of cross-over experiment

This appendix presents the information provided to participants in the cross-over experiment. For six table–graph pairs we created a brief *textual summary* and *question set*. These are presented on the pages that follow. Following each question set are corresponding figures. For reasons of copyright, we do not present the tables used; these may be found in the original publications.

Generalised thresholding estimators for high dimensional location parameters

Scenario

Zhang et al (2010, *Statistica Sinica* 20:911–26) introduced the use of generalised thresholding estimators to estimate high dimensional location parameters when analyzing high-throughput genomic, proteomic and metabolomic data. They considered estimating a high dimensional location parameter $\mu = (\mu_1, \mu_2, \ldots, \mu_p)$ from noisy data $Y_p = (y_1, y_2, \ldots, y_p)$ where

$$y_i - \mu_i \overset{\text{iid}}{\sim} \varphi(.)$$

where $\varphi(.)$ is a symmetric log-concave density function.

Estimators and parameters

They conducted a simulation study to compare their generalised empirical Bayes (GEB) estimators with the following five estimators:

a) Empirical Bayes estimator (EB),

b) Stein's unbiased risk estimator (SURE), and

c) Estimators controlling the False Discovery Rate (FDR) for three types of risk, q:

- FDR ($q = 0.01$),
- FDR ($q = 0.1$), and
- FDR ($q = 0.4$).

They investigated in a simulation study the performance of these estimators for several combinations of the location parameters μ_i (equal to μ_- or μ_+) and the number of negative and positive parameters, k_- and k_+. Here the focus is on the case of $|\mu_-| = \mu_+$ but $k_- \neq k_+$ in the [**plots/table**] below.

Criteria

The performance of the estimators was assessed by three criteria:

a) the risk level, $R(2)$,

b) the number of false positives, NFP, and

c) the number of false negatives, NFN.

For all criteria, a large value indicates poor performance. Note there is a partial trade-off between the number of false positives and negatives.

Questions

1. For which value of μ_-/μ_+ does FDR $(q = 0.01)$ give the smallest risk, $R(2)$, for all levels of k_-/k_+?
2. FDR can control the number of false positives very well but may report a large number of false negatives for an inappropriate choice of q. For what choice of q does the FDR estimator usually have a larger number of false negatives than GEB?
3. Refer to the NFP criterion. For what level of k_-/k_+ is the estimator EB worse than the estimator SURE?
4. Which estimator almost always gives one of the smallest NFNs but almost always the largest NFPs?
5. Which estimator usually gives the largest NFNs?

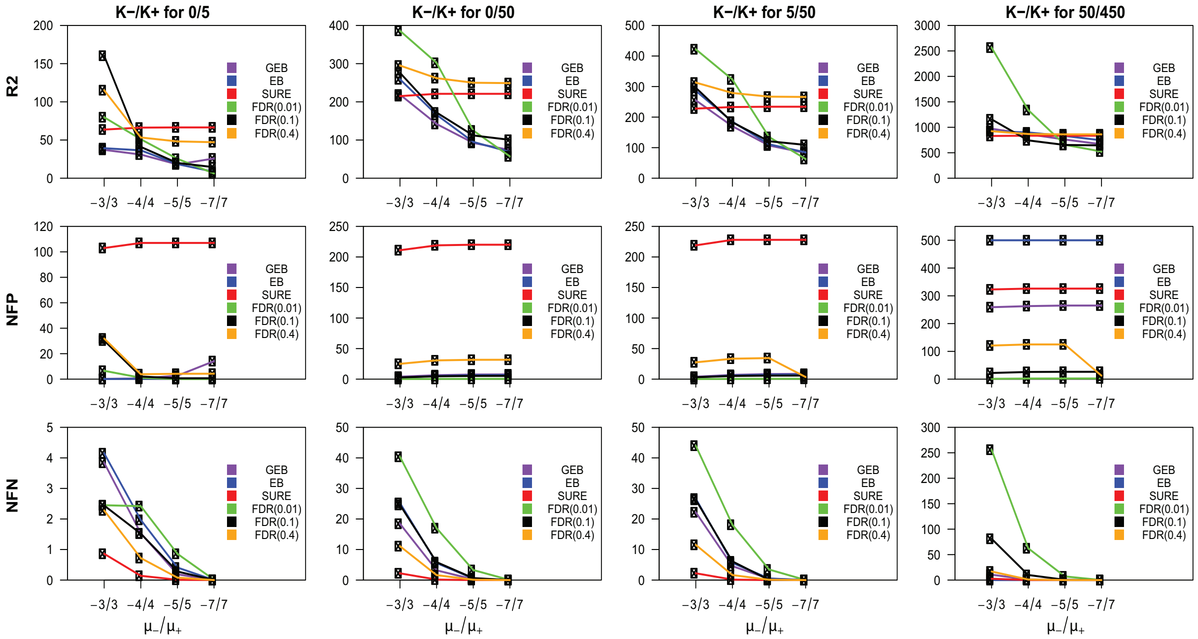

Iterated smoothed bootstrap confidence intervals for population quantiles

Scenario

Ho and Lee (2005, *Annals of Statistics* 33:437–62) investigated the effects of smoothed bootstrap iterations on coverage probabilities of smoothed bootstrap and bootstrap-t confidence intervals for population quantiles.
They proposed two methods:

- an iterated smoothed bootstrap t method, and
- an iterated smoothed bootstrap percentile method,

and conducted a simulation study to investigate the effects of the iterated smooth bootstrap on coverage probabilities of both one- and two-sided confidence intervals.

Distributions

1 000 random samples of size n were generated from three underlying distributions:

- the standard normal distribution, $X \sim \mathrm{N}(0, 1)$,
- a double exponential distribution, $f(x) = e^{-|x|}/2$, and
- the standard lognormal distribution, $X \sim \log \mathrm{N}(0, 1)$.

Sample Sizes

Three sample sizes were considered:

- $n = 15$,
- $n = 30$, and
- $n = 100$.

Types of intervals

Seven methods to derive intervals were considered:

- $\mathrm{I}_{1,\alpha}$, a non-iterated smoothed bootstrap percentile upper confidence interval,
- $\mathrm{I}_{2,\alpha}$, an iterated smoothed bootstrap percentile upper confidence interval,
- $\mathrm{I}_{3,\alpha}$, a smoothed bootstrap-t upper confidence interval,
- $\mathrm{I}_{4,\alpha}$, an iterated smoothed bootstrap-t upper confidence interval,
- $\mathrm{I}_{BH,\alpha}$, Beran and Hall's interpolated interval,
- $\mathrm{I}_{EL,\alpha}$, Chen and Hall's smoothed empirical likelihood interval, and
- $\mathrm{I}_{EL(B),\alpha}$, Chen and Hall's smoothed empirical likelihood intervals (Bartlett corrected).

Criteria

They assess three coverage probabilities:

- Lower: 5%,
- Upper: 95%, and
- Overall: 90% two sided interval $I^2_{j,0.9}$.

The following [**plots/table**] show the estimated coverage probabilities of the variously derived intervals, denoted $\mathrm{I}_{j,\alpha}$ for criteria lower, upper and overall, for $j = 1, 2, 3, 4, BH, EL$, and $EL(B)$.

Questions

1. Which method has the least accurate estimated coverage probability in all cases considered?
2. For which sample sizes does $I_{BH,\alpha}$ have the most accurate estimated overall coverage probability?
3. Which interval most often gives a coverage probability most close to the advertised coverage for small samples?
4. For standard normal data, does $I_{4,\alpha}$ give its most accurate estimated coverage probability for (i) the lower tail (ii) the upper tail or (iii) overall?
5. For which distribution does the overall coverage probability display the greatest variability around the advertised coverage across the different methods?

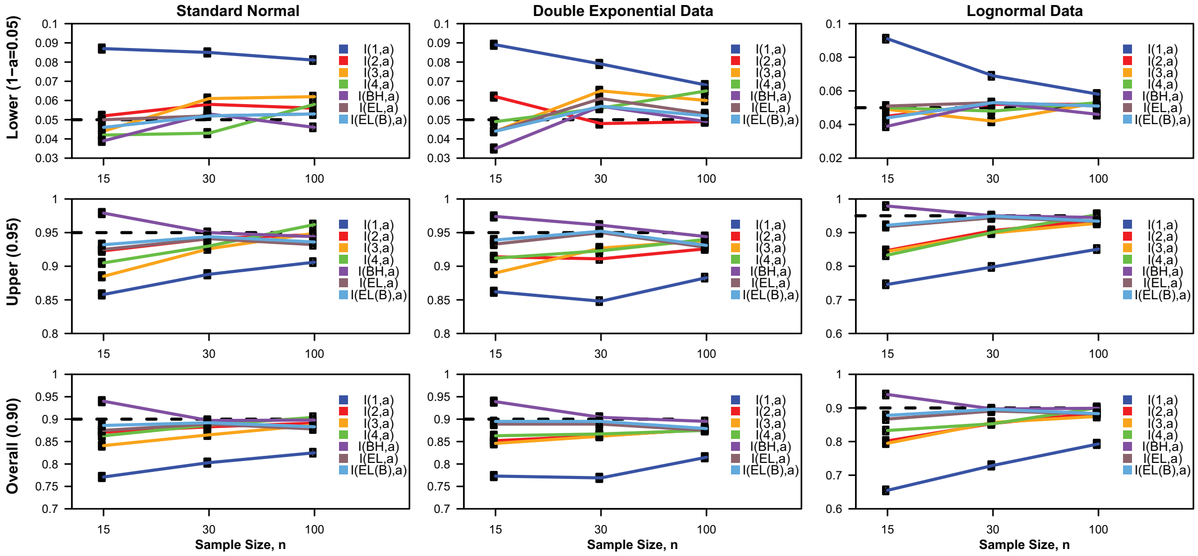

Confidence intervals for the long memory parameter based on wavelets and resampling

Scenario

Conti et al (2008, *Statistica Sinica* 85:537–43) studied the problem of constructing confidence intervals for the long memory parameter of stationary Gaussian processes with long-range dependence. They performed a simulation study in order to evaluate the actual coverage probabilities of confidence intervals derived from different mother wavelets.

Parameters

They considered two values of H, the Hurst self-similarity parameter:

- $H = 0.6$, and
- $H = 0.8$.

Sample Sizes

Two sample sizes were used in the simulations:

- $n = 2^{11}$ (i.e. around 2 000), and
- $n = 2^{15}$ (i.e. around 30 000).

Criteria

Three types of mother wavelets were used:

- Haar,
- Daubechies 2, and
- Daubechies 4.

The following [**plots/table**] shows the coverage probabilities of confidence intervals for the Hurst parameter, H.

Questions

1. Overall, for which of the two values of H do all three wavelets have a coverage probability furthest from the advertised coverage probability?
2. When the target is to get 95% coverage, which wavelet has the worst performance, i.e. the coverage furthest from 95%?
3. Consider the case when $H = 0.6$. For which block size does Daubechies 4 give the best coverage for Normal Level of 0.99?
4. For a block size of 2^8, for which nominal coverage level does HAAR perform worse than the other two wavelets?
5. Overall, which wavelet performs best for large block sizes of 2^9?

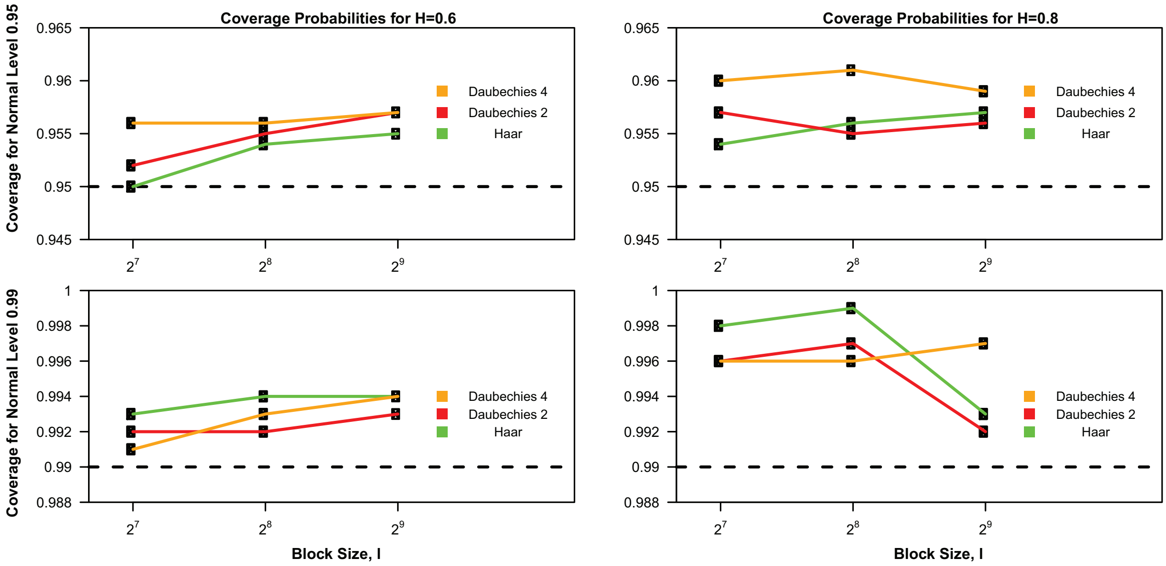

Bootstrap calibration for confidence interval construction and selection

Scenario

Loh (1991, *Statistica Sinica* 1:477–491) proposes bootstrap calibration as a method selection tool for interval construction with different orders of asymptotic error rate instead of the intervals obtained from the conventional Bootstrap t method.
He derived a new bootstrap recipe and studied its asymptotic properties for one term Edgeworth-corrected confidence bounds in the more general context of estimating a smooth function of vector means, and performed a simulation experiment to compare four calibrated intervals with the Bootstrap t interval, for finite sample sizes.

Sample Sizes

Three sample sizes (n) were considered:

- $n = 10$,
- $n = 25$, and
- $n = 50$.

Distributions

Two distributions for data were used, namely:

- the normal distribution, and
- the exponential distribution.

Criteria

Three types of intervals were found:

- 95% left-closed right-open,
- 95% left-open right-closed, and
- 90% two sided.

Intervals

Four calibrated intervals based upon

- normal theory,
- Johnson t,
- approximated calibrated normal theory, and
- approximated calibrated Johnson t

were compared against bootstrap t intervals for the one-sample mean problem.
The following [**plots/table**] shows the estimated coverage probabilities of the five intervals.

Questions

1. In what situation does a bootstrap t interval give the best coverage probability over all sample sizes?
2. In what situation does a bootstrap t interval give the worst coverage probability over all sample sizes?
3. Which interval gives the best coverage probabilities for a one sided, exponential distribution in general?
4. For normal data, for which sample size do all intervals have almost the same coverage probabilities?
5. Which interval is closest to the bootstrap t interval for normally distributed data?

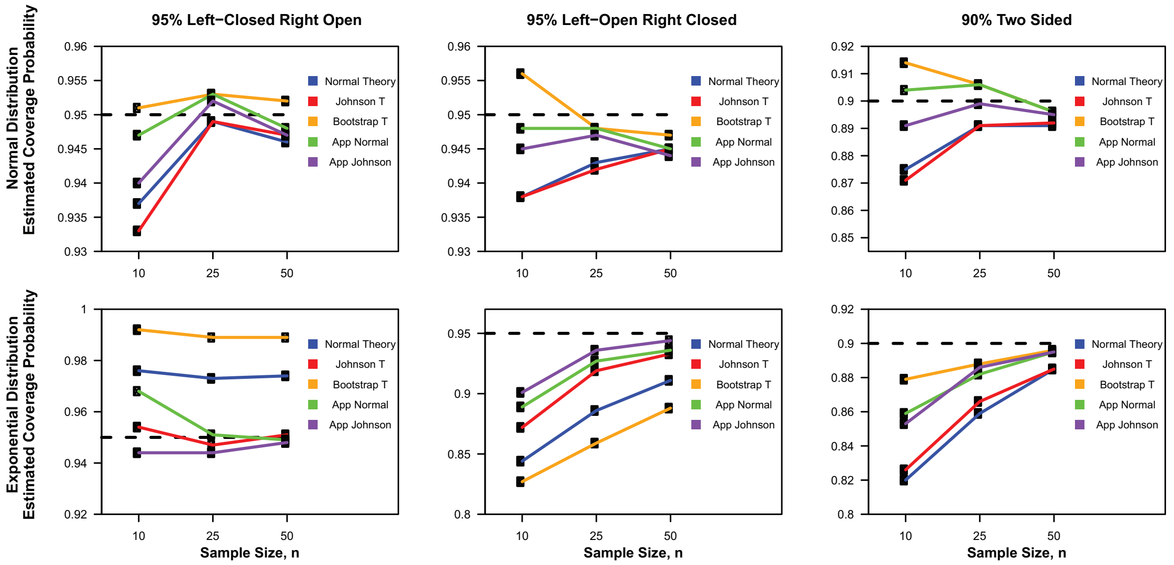

Testing for trend in the presence of autoregressive error

Scenario

Roy et al (2004, *J Am Stat Assoc* 99:1082–91) describe various methods to perform null hypothesis significance tests of the hypothesis of no linear trend in autoregressive time series. The model they consider is:

$$\begin{aligned} Y_t &= \mu + \beta t + y_t \\ y_t &= \alpha y_{t-1} + \psi_1 \Delta y_{t-1} + ... + \psi_{p-1} \Delta y_{t-p+1} + e_t \end{aligned}$$

where e_t are *iid* with mean 0 and variance σ^2, and $t = 1, \ldots, T$.

Criteria

They compare the empirical power of the following methods:

a) feasible generalised least squares (FGLS) 1: this test statistic uses the median unbiased estimator of α to test the hypothesis of β=0;

b) FGLS2: this uses a "positively biased estimator" of α;

c) pretest 1A: this involves first testing the hypothesis α=1 and if it is not rejected (at the 2.5% level) testing β=0 using the mean of the differences, whereas if it is rejected, using the WSLS estimator;

d) pretest 1B: is the same as pretest 1A but uses the FGLS test if α=1 is rejected;

e) Gauss–Newton: involves taking Taylor expansions around an initial (biased) estimator of the parameter vector.

They perform a simulation study with 10 000 simulations and a sample size of 100, and for a range of values of α calculate the empirical power of these tests. Results are presented in the following [**plots/table**].

Questions

1. Which is the most powerful test when the autoregressive parameter α is greater than 0.90?
2. Which is the most powerful test when α is less than 0.90?
3. Which test usually has the lowest power?
4. Which three tests are *almost* identical in empirical power for $0 < \alpha < 0.60$?
5. For which values of α is Pretest1B more powerful than FGLS1?

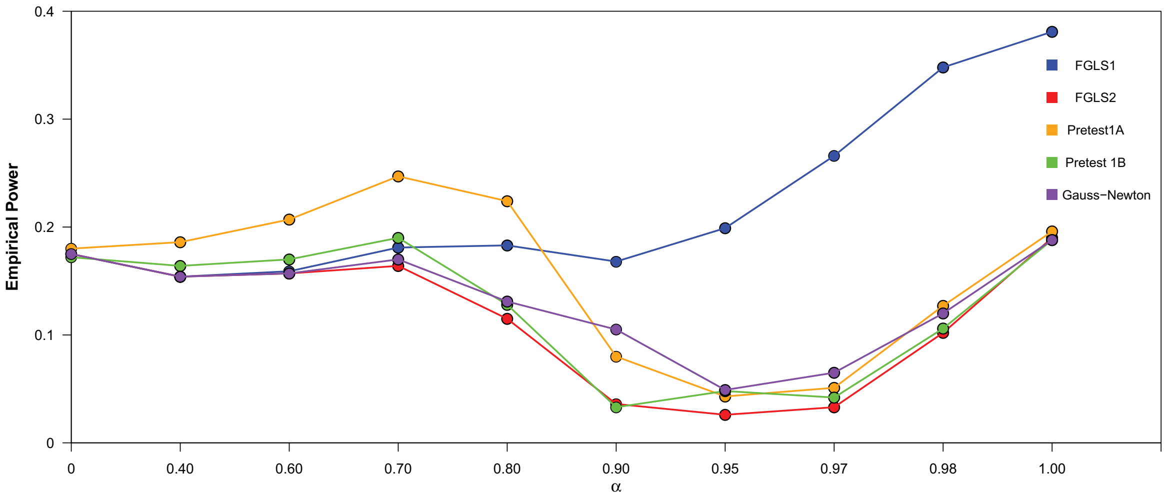

Empirical Power of Tests for the Trend coefficient at 1 SD away. (T=100 ; 10000 samples)

Efficiencies of Methods Dealing with Missing Covariates in Regression Analysis

Scenario

Wang and Paik (2006, *Statistica Sinica* 16:1169–92) compare the efficiencies of various approaches that have been used to deal with the problem of missing covariates in regression analysis. They performed simulation studies with 500 replications to examine the performances of non-likelihood approaches. Two models—the classical linear model and the logistic linear model—were considered, and here we focus on the logistic linear model:

$$P(Y = 1|Z, X, \beta) = \text{logit}^{-1}(\beta_0 + \beta_z Z + \beta_x X)$$

where

- X is a binary variable with $P(X = 1) = 0.5$,
- Z is a binary variable with $P(Z = 1) = 0.5$, and
- $\beta = (\beta_0, \beta_z, \beta_x) = (-0.5, \log 2, \log 2)$

Categories, Sample Size and Parameters

There are two categories of missing mechanisms:

- MCAR: Missing Completely at Random, and
- MAR: Missing at Random,

using logistic models for the probability of being observed that may be a function of the covariate Z and the outcome Y.
Three parameters were to be estimated:

- β_0,
- β_z, and
- β_x,

for two sample sizes:

- $n = 100$ and
- $n = 500$.

Criteria

Four measures of performance were used:

- Bias: the Monte Carlo mean (over 500 simulations) difference between the true value and the estimator;
- SD: the Monte Carlo standard deviation of the estimator;
- MSD: the estimator's mean estimated standard error;
- CP: the coverage probability of 95% confidence intervals.

Approaches

A total of 11 approaches were evaluated:

- Full: maximum likelihood estimates from the full data,
- CC: complete case analysis,
- IPWt: inverse probability weighting estimates using the true observation probability, $\pi(Y, Z)$,
- IPW0: inverse probability weighting estimates from model 0 (only Z influences the observation probability),
- IPW1: inverse probability weighting estimates from model 1 (Z and Y have main effects on the observation probability),
- IPW2: inverse probability weighting estimates from model 2 (Z, Y and their interaction influence the observation probability),
- Cont: conditional estimates using the true observation probability, $\pi(Y, Z)$,
- Con1: conditional estimates from model 1 (i.e. Z and Y main effects),
- Con2: conditional estimates from model 2 (i.e. Z, Y and their interaction),
- IPWff: improved augmented IPW estimates, and
- Impu: imputation estimates.

Three sets of [**plots/table**] are presented below, one per parameter being estimated (β_0, β_z, and β_x). Each set shows rankings for each approach within each performance measure (e.g., bias). The higher the ranks, the better the approach performs.

Questions:

1. Refer to the output relating to β_0. Rank IPW0, IPW1, IPW2 and IPWT in terms of their standard deviation SD, from the best to the worst.

2. When estimating β_x, which sample size leads to IPW0 performing better for the bias measure?

3. For which β values does IMPU perform better than IPW1 in having a low SD?

4. Under which category of missing mechanisms do all parameters have very similar performances for the CP of β_0?

5. Which are the three approaches with the worst MSD for β_z?

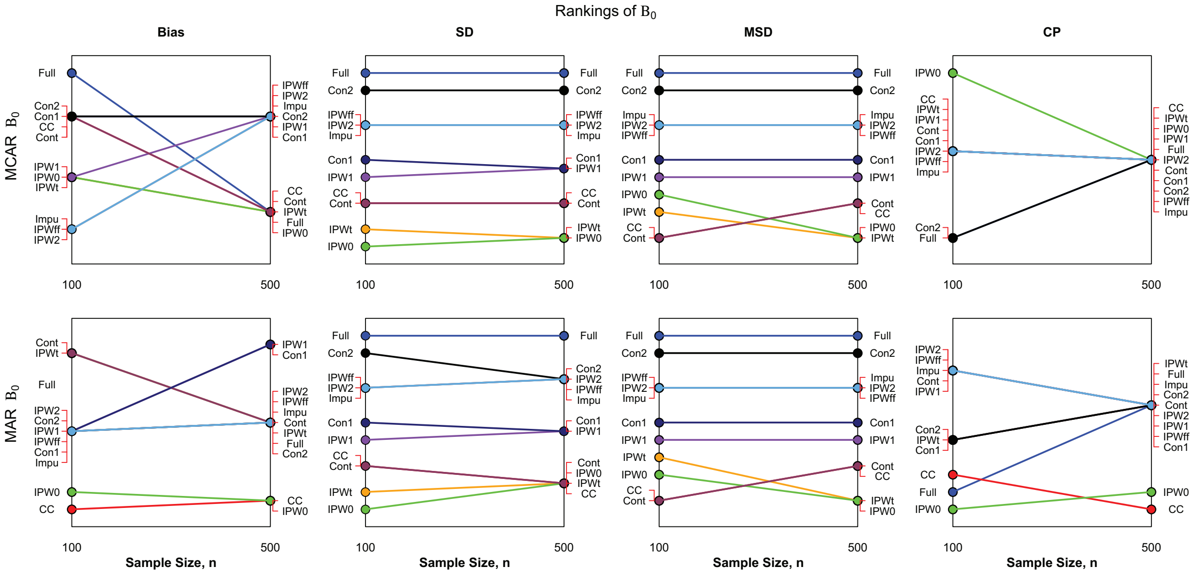

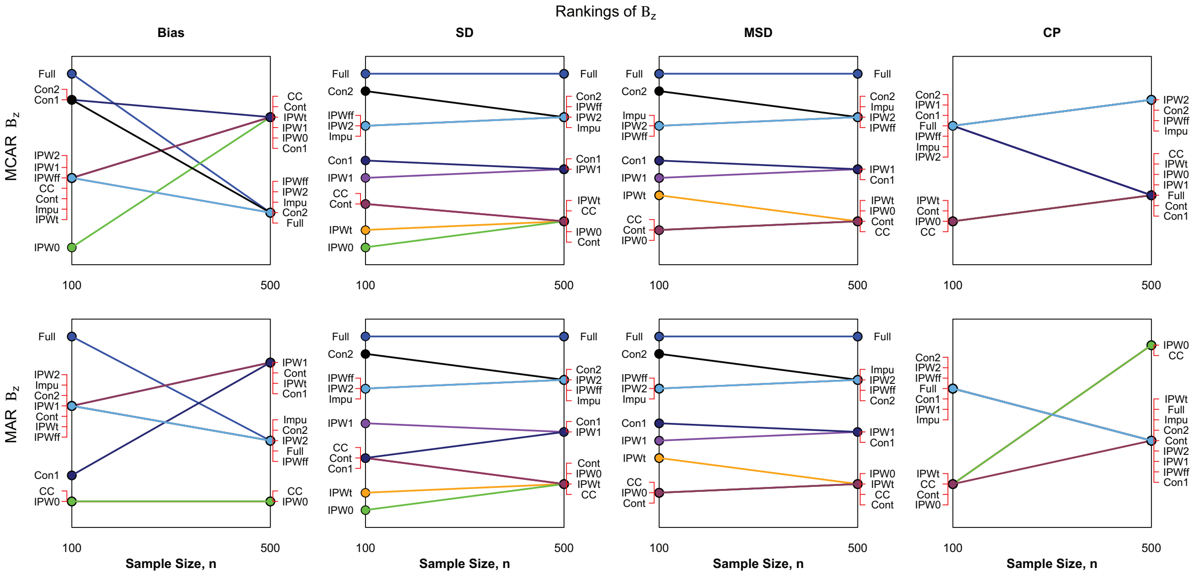

Rankings of B_z
Bias
SD
MSD
CP
MCAR B_z
MAR B_z
100
500
Sample Size, n
Full
Con2
Con1
Cont
CC
Impu
IPWt
IPW0
IPW1
IPW2
IPWff

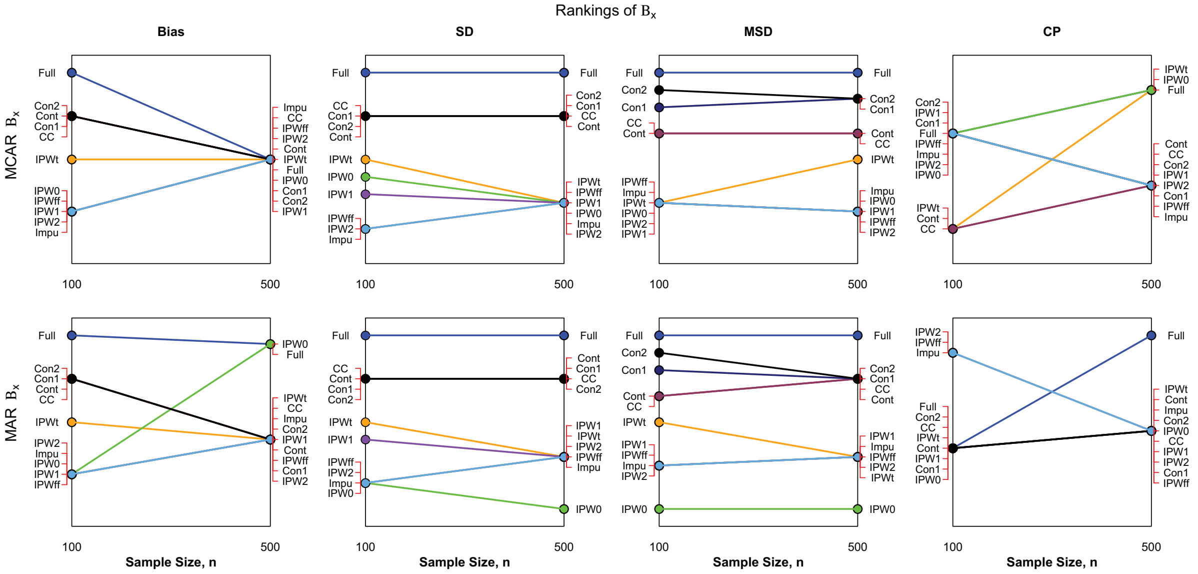

Rankings of B_x
Bias
SD
MSD
CP
MCAR B_x
MAR B_x
100
500
Sample Size, n
Full
Con2
Con1
Cont
CC
Impu
IPWt
IPW0
IPW1
IPW2
IPWff

Appendix 2: full specification of models used

The data-parameter model for the time taken to answer questions is specified by:

$$
\begin{aligned}
T_{ijk} &\sim \log \mathrm{N}(\mu_{ijk}, \sigma^2) \\
\mu_{ijk} &= \gamma + \alpha_i + \beta_{Q_{ij}} + \delta_{ij} \\
\gamma &\sim \mathrm{U}(-100, 100) \\
\alpha_i &\sim \mathrm{N}(\mu_i, \sigma_\alpha^2) \\
\mu_i &= 0 \text{ if } E_i = 0 \\
&= \mu_\alpha \text{ if } E_i = 1 \\
\mu_\alpha &\sim \mathrm{U}(-100, 100) \\
\sigma_\alpha &\sim \mathrm{U}(0, 100) \\
\beta_j &\sim \mathrm{N}(0, \sigma_\beta^2) \\
\sigma_\beta &\sim \mathrm{U}(0, 100) \\
\delta_{ij} &= \delta_1 = \theta_1 \text{ if } E_i = 0 \text{ and } j = 1 \\
&= \delta_2 = \theta_1 + \theta_2 \text{ if } E_i = 1 \text{ and } j = 1 \\
&= 0 \text{ if } j = 0 \\
\theta_1 &\sim \mathrm{U}(-100, 100) \\
\theta_2 &\sim \mathrm{U}(-100, 100) \\
\sigma &\sim \mathrm{U}(0, 100)
\end{aligned}
$$

where

- T_{ijk} is the time taken by the ith individual to answer her or his kth question on treatment arm j, for $k \in \{1, \ldots, 5\}$, $j \in \{0, 1\}$, and $i \in \{1, \ldots, 20\}$;
- E_i is the experience level of individual i, with $E_i = 0$ for undergraduate participants and $= 1$ for postgraduates;
- $Q_{ij} \in \{1, \ldots, 6\}$ indexes the table or graph.

For accuracy, the model becomes:

$$
\begin{aligned}
A_{ijk} &\sim \text{Bernoulli}(p_{ijk}) \\
\text{logit}(p_{ijk}) &= \gamma + \alpha_i + \beta_{Q_{ij}} + \delta_{ij} \\
\gamma &\sim \text{U}(-100, 100) \\
\alpha_i &\sim \text{N}(\mu_i, \sigma_\alpha^2) \\
\mu_i &= 0 \text{ if } E_i = 0 \\
&= \mu_\alpha \text{ if } E_i = 1 \\
\mu_\alpha &\sim \text{U}(-100, 100) \\
\sigma_\alpha &\sim \text{U}(0, 100) \\
\beta_j &\sim \text{N}(0, \sigma_\beta^2) \\
\sigma_\beta &\sim \text{U}(0, 100) \\
\delta_{ij} &= \delta_1 = \theta_1 \text{ if } E_i = 0 \text{ and } j = 1 \\
&= \delta_2 = \theta_1 + \theta_2 \text{ if } E_i = 1 \text{ and } j = 1 \\
&= 0 \text{ if } j = 0 \\
\theta_1 &\sim \text{U}(-100, 100) \\
\theta_2 &\sim \text{U}(-100, 100)
\end{aligned}
$$

where $A_{ijk} = 1$ if the ith individual answered her or his kth question on treatment arm j correctly and 0 if not.
